# Fine Particulate Matter and Parkinson Disease Risk Among Medicare Beneficiaries

**DOI:** 10.1212/WNL.0000000000207871

**Published:** 2023-11-21

**Authors:** Brittany Krzyzanowski, Susan Searles Nielsen, Jay R. Turner, Brad A. Racette

**Affiliations:** From the Barrow Neurological Institute (B.K., B.A.R.), Phoenix, AZ; Washington University in St. Louis (S.S.N., J.R.T.) MO.

## Abstract

**Background and Objectives:**

Numerous studies suggest that environmental exposures play a critical role in Parkinson disease (PD) pathogenesis, and large, population-based studies have the potential to advance substantially the identification of novel PD risk factors. We sought to study the nationwide geographic relationship between PD and air pollution, specifically PM_2.5_ (particulate matter with a diameter <2.5 micrometers), using population-based US Medicare data.

**Methods:**

We conducted a population-based geographic study of Medicare beneficiaries aged 66–90 years geocoded to US counties and zip+4. We used integrated nested Laplace approximation to create age, sex, race, smoking, and health care utilization–adjusted relative risk (RR) at the county level for geographic analyses with PM_2.5_ as the primary exposure of interest. We also performed an individual-level analysis using logistic regression with cases and controls with zip+4 centroid PM_2.5_. We adjusted a priori for the same covariates and verified no confounding by indicators of socioeconomic status or neurologist density.

**Results:**

Among 21,639,190 Medicare beneficiaries, 89,390 had incident PD in 2009. There was a nationwide association between average annual PM_2.5_ and PD risk whereby the RR of PD was 56% (95% CI 47%–66%) greater for those exposed to the median level of PM_2.5_ compared with those with the lowest level of PM_2.5_. This association was linear up to 13 μg/m^3^ corresponding to a 4.2% (95% CI 3.7%–4.8%) greater risk of PD for each additional μg/m^3^ of PM_2.5_ (*p*_trend_ < 0.0001). We identified a region with high PD risk in the Mississippi-Ohio River Valley, where the risk of PD was 19% greater compared with the rest of the nation. The strongest association between PM_2.5_ and PD was found in a region with low PD risk in the Rocky Mountains. PM_2.5_ was also associated with PD in the Mississippi-Ohio River Valley where the association was relatively weaker, due to a possible ceiling effect at average annual PM_2.5_ levels of ∼13 μg/m^3^.

**Discussion:**

State-of-the-art geographic analytic techniques revealed an association between PM_2.5_ and PD that varied in strength by region. A deeper investigation into the specific subfractions of PM_2.5_ may provide additional insight into regional variability in the PM_2.5_-PD association.

## Introduction

Several studies have linked air pollution in the form of aerosolized particulate matter to various adverse health outcomes. Recent investigations identified associations between fine particulate matter, that is, particulate matter with diameter ≤2.5 micrometers (PM_2.5_), and neurologic disease, including dementia^1^ and stroke.^2^ The ultrafine particles (≤0.1 micrometers) in PM_2.5_ cross the blood-brain barrier in humans.^3^ In addition, some subcomponents of PM_2.5_ are more neurotoxic than others. In particular, PM_2.5_ can contain heavy metals, including arsenic and manganese, which have been implicated in the neuropathogenesis of basal ganglia degeneration.^4,5^ Despite this, epidemiologic investigations of PM_2.5_ and Parkinson disease (PD) have yielded mixed results,^6-20^ with marked discrepancies in the magnitude, shape, and even direction of PM_2.5_-PD associations. One possible contributor is the use of different PD-related outcomes and widely varying definitions of “incidence.” In addition, the range of PM_2.5_ levels across studies varies widely, and differences in the size and content of particulate matter in different regions might influence PM_2.5_-PD associations. Accordingly, several prior studies found PM_2.5_-PD associations to differ by geographic area^10-12^ or urban/rural land use.^16,18^ Several powerful spatial analytic methods offer to advance our understanding of the role of PM_2.5_ in PD by enabling high-resolution, population-based investigations of the geographic distribution of PD, to characterize patterns of incidence and their relation to PM_2.5_ exposure.

Similar to studies of PM_2.5_ and PD, existing research into the national distribution of PD primarily consists of studies of mortality and prevalence.^21-23^ One study found a North-South gradient in PD mortality and prevalence, which likely reflects the general burden of disease but does not capture the geographic patterns of *incidence* necessary for understanding the role of environmental risk factors. In addition, most nationwide studies of PD rely on state-level data.^22,24^ To date, only one nationwide, county-level study of incident PD has been conducted in the United States.^21^ This study found nonrandom clustering of PD in the Midwest and East South Central United States, which likely reflects, in part, the effect of environmental exposures on PD risk. Deeper exploration of these PD clusters, and the potential role of PM_2.5_ in contributing to those clusters, requires further investigation with advanced geographic methods and geostatistical approaches not yet applied to neurodegenerative disease. We conducted a US population-based geographic study of PD risk to examine spatial patterns of newly diagnosed PD and relationships with PM_2.5_, using a multimethod approach that included spatial analytic and statistical methods. We hypothesized that we would identify spatial clustering of PD and observe a positive association between PM_2.5_ and PD, which would vary by region.

## Methods

This study was approved by the Human Research Protection Office at Washington University School of Medicine in St. Louis and by the Centers for Medicare & Medicaid Services. Participant consent in this records-based study was not required.

### Study Population and Case Ascertainment

Our eligibility criteria were designed to ensure a population-based sample with complete data: age-eligible for Medicare ≥2 years before diagnosis/selection (66 years and older), no Part C (Medicare Advantage/health maintenance organization) coverage, 90 years and younger, and US residence, all in 2009, without additional (e.g., medical) inclusion/exclusion criteria.^25^ Incident PD cases included all study-eligible beneficiaries with at least one International Classification of Diseases, Ninth Revision, diagnosis code of 332 or 332.0 in 2009, but no prior year. This case definition, similar to a few prior studies of PM_2.5_ and PD,^17,19,20^ which maximizes sensitivity without materially affecting specificity,^26^ aims to ensure representativeness of cases, including with regard to exposures. Beneficiaries with a diagnosis of atypical parkinsonism (333.0) or Lewy body dementia (331.82) were excluded if diagnosed in the year of PD diagnosis or earlier (4.6% potential cases).^25^ We geocoded beneficiaries to their residential zip+4 after applying a two-year lag in their residential history, which we obtained from the 2007 Medicare beneficiary annual summary file. For maps and geographic analyses, we linked beneficiaries to county of residence to avoid zero inflation.

### PM_2.5_ Exposure Estimation

Our exposure of a priori interest was average annual PM_2.5_ from 1998 to 2000,^27^ a period largely before PD onset. We used this period because it is likely etiologically relevant and 90% of beneficiaries maintained the same county code (84% had the same 5-digit zip code) for all years available (2004–2009). PM_2.5_ data,^27^ which were available in 1-kilometer grids, were based on several predictors, including satellite, meteorologic, land use, and elevation data with varying resolutions.^28^ This PM_2.5_ model achieved a cross-validated R^2^ of 0.89. All 3 prior PD studies that used this or a similar PM_2.5_ model^7,16,17^ observed significant associations between PM_2.5_ and PD, consistent with an acceptable degree of exposure measurement error in the context of air pollution health effects research, where associations are generally difficult to detect. We then used geographic information systems to estimate average annual PM_2.5_ at 2 different geographic levels: (1) county for mapping and spatial analyses and (2) zip+4 (centroid) for individual-level regression analysis.

### Assessment of Covariates

For all essential covariate data, we started with data at the individual (beneficiary) level and only collapsed to the county level for mapping and spatial analyses. Covariates included beneficiary demographic information (age, sex, and race) and measures of health care utilization in the year before diagnosis/reference from the beneficiary annual summary file. We defined health care utilization as the number of physician visits (carrier) and outpatient visits either for the individual or for county-level analyses per county for all Medicare beneficiaries in our study. We obtained the county-level current prevalence of smoking cigarettes^29^ for county-level/geographic analyses and estimated the probability of ever smoking at the individual level for individual-level analyses. We developed this smoking variable for use in geographic studies through a multivariable linear regression model with our validated claims-based probability of smoking^30^ as the outcome. This gold-standard variable replicates the well-established relationship between smoking and PD.^25^ Predictors of smoking probability in the new model were county-level prevalence of current smoking^29^ and individual-level data from the beneficiary annual summary file. Similar to the original smoking model based on detailed claims, these individual-level predictors included sex, race, birth cohort, and selected medical conditions—here chronic obstructive pulmonary disease, lung cancer, stroke, acute myocardial infarction, other ischemic cardiovascular disease, stroke/transient ischemic attack, chronic kidney disease, osteoporosis, and depression. This smoking variable along with use of care, age, sex, and race represented our core set of covariates. As additional covariates for sensitivity analyses, we obtained the following ecologic data: census tract airborne trichloroethylene,^31^ county-level area deprivation,^32^ census block group median household income,^33^ neurologists per county per 100,000 Medicare beneficiaries,^21^ and county-level agricultural pesticide use.^34^ In addition, from the beneficiary annual summary file, we obtained individual-level data on acute myocardial infarction, ischemic heart disease, stroke/transient ischemic attack, congestive heart failure, chronic obstructive pulmonary disease, and diabetes. These 6 conditions might be caused by PM_2.5_ exposure and subsequently lead to care that facilitates diagnosis of PD, that is, act as nuisance mediators.

### Estimation of PD Relative Risk for Counties

We used integrated nested Laplace approximation for Bayesian inference in R-Project (R-INLA)^35^ using R version 4.1.2 to estimate county-PD relative risks (RR) that account for known demographic risk factors^36-40^ and spatial dependency. Specifically, these R-INLA–derived RRs were based on Gaussian distribution using indirect age-sex-race standardized incidence ratios, health care utilization, and smoking as input. We calculated the standardized incidence ratio by dividing the number of observed cases by expected counts and then multiplying by 100, based on 4 age (65–69, 70–74, 75–79, and 80+ years), sex, and 5 race (White, Black, Asian, Hispanic, and other) strata. To address spatial autocorrelation, we integrated spatial dependency into the R-INLA model using conditional autoregressive distribution to smooth risks according to the standardized incidence ratios of neighboring counties. The R-INLA–derived PD RR for each county was available for all county-level analyses.

### Assessment of Spatial Clustering of PD

To formally test for spatial clustering and identify high and low PD risk counties, we used univariate local indicators of spatial association (LISAs) to map PD hot and cold spots.^41^ Hot-spot counties have above-average PD risk (RR) and share boundaries with counties that all have above-average PD risk. Cold-spot counties have below-average PD risk and share boundaries with counties that have below-average PD risk. LISAs identify where high and low-risk counties form contiguous clusters rather than only focusing on counties with significant RRs. LISAs also provide *Global Moran's I* value to describe the nationwide presence or absence of clustering.

### Examination of the PM_2.5_-PD Association

We used 3 approaches to examine the relationship between PM_2.5_ and PD—a regression model for assessing the *nationwide* relationship and 2 geographic approaches for assessing *regional* relationships. Specifically, for our nationwide assessment, we performed traditional multivariable regression at the individual level using Stata/MP version 17. For regional assessment, to explore whether the PM._2.5_-PD association differed by region, we used bivariate LISAs implemented in GeoDa version 1.20^41^ and geographically weighted regression (GWR) implemented in ArcGIS. Bivariate LISAs were used to assess and visualize local spatial correlation between PD and PM_2.5_ while GWR was used to determine and quantify the *direction and strength* of local associations.

#### Logistic Regression Analysis

We performed logistic regression with PD as the outcome and zip+4 PM_2.5_ as the independent variable, adjusted for the same a priori covariates as above but assessed at the individual level. To initially examine the association while allowing for nonlinear associations, we modeled PM_2.5_ as deciles, with the lowest decile of PM_2.5_ as the reference group. Based on these results, we then sought to develop a more parsimonious model using linear splines. We selected the final model using the Akaike information criterion while also examining the sensitivity of results to knot number and placement. To assess whether restriction to Medicare-aged beneficiaries could limit generalizability, we tested whether the PM_2.5_-PD association differed by age. To assess whether associations might be due to occupational rather than environmental exposures, we tested whether the association was stronger in men than women. We tested for interaction on the multiplicative scale while including main-effects terms in the model. As a sensitivity analysis, we included Lewy body dementia cases. Because PD is relatively rare, the odds ratio provides an accurate estimate of the RR.

#### Spatial Correlation

We used bivariate LISAs to overlay PM_2.5_ and PD hot and cold spots and assess local spatial correlation.^41^ A bivariate LISA map is the convergence of 2 univariate LISA maps into a single map (i.e., a map of PD hot and cold spots + a map of PM_2.5_ hot and cold spots). A bivariate LISA map delineates 4 cluster categories (high-high, low-low, low-high, and high-low). The high-high category represents counties where PD risk and PM_2.5_ levels are both high relative to their means, and the low-low category represents counties where PD risk and PM_2.5_ levels are both low; these categories are consistent with a positive correlation between PM_2.5_ and PD. The 2 discordant (low-high and high-low) categories are inconsistent with a positive correlation.

#### Spatial Regression

We used GWR to determine the *direction and strength* of the local associations between PM_2.5_ and PD (ArcGIS). In GWR, local regression is performed for each county using surrounding counties. We used an adaptive spatial bandwidth to define the latter. GWR computed a regression coefficient for each county using county-PD RR as the dependent variable and PM_2.5_ as the independent variable. We retained RR as a continuous outcome and modeled PM_2.5_ in deciles in a single continuous variable. Thus, the GWR beta coefficients represent the absolute difference in the PD RR when going from any decile of PM_2.5_ to the decile below. We then computed 95% confidence intervals (CIs) and mapped the GWR coefficients for counties where the CI excluded zero (significant at a two-sided alpha = 0.05). This allowed us to include both the positive and negative coefficients in a map, as well as to produce a map focused on regions where GWR coefficients were positive, providing a more conservative reflection of where the association between PM_2.5_ and PD was positive. In addition, we used the Monte Carlo test of spatial variability to assess objectively whether the relationship between PM_2.5_ and PD varied across the nation.

### Data Availability

The Centers for Medicare & Medicaid Services does not permit data sharing under the data use agreement.

## Results

After excluding prevalent PD, 21,639,190 US Medicare beneficiaries from Medicare research files^42^ met initial study eligibility criteria,^43^ including 89,790 PD incident cases and 21,549,400 non-cases. Of these, we had 65,180 cases (73%) and 15,561,435 non-cases (72%) with high-resolution residence information available (zip+4) 2 years before PD diagnosis or the control reference date. We were restricted to those with zip+4 information so that our ecologic exposure estimates would better represent individual-level exposures. We observed no demographic differences between the full data set^43^ vs this sample (Table 1), overall or by case status. In both samples, 89% of cases and 86% of controls were non-Hispanic White and 6% of cases and 8% of controls were Black. Sex and age distribution for cases and controls were essentially identical between the full and present sample as well.

**Table 1:**
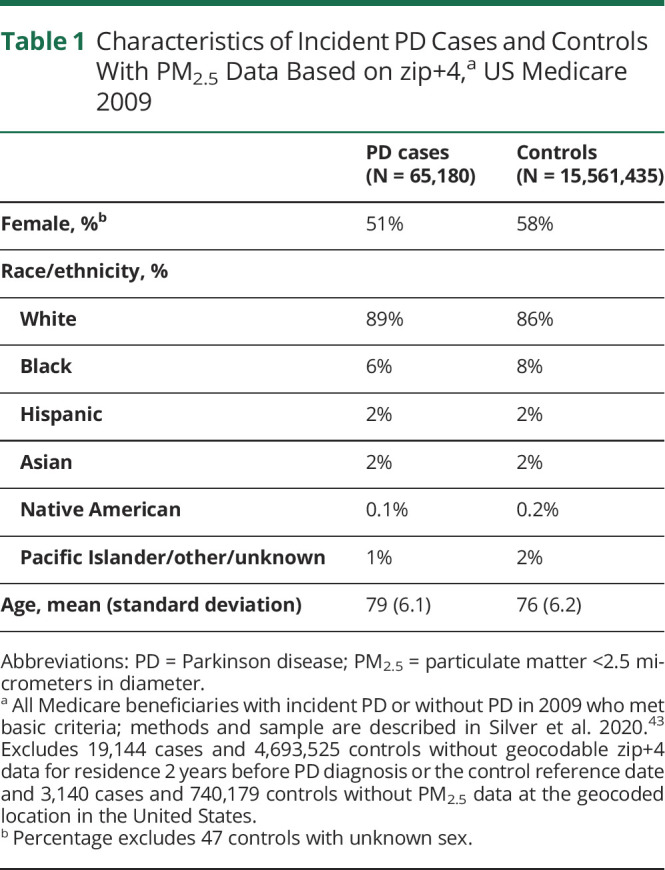
Characteristics of Incident PD Cases and Controls With PM_2.5_ Data Based on zip+4,^a^ US Medicare 2009

	PD cases (N = 65,180)	Controls (N = 15,561,435)
Female, %^b^	51%	58%
Race/ethnicity, %		
White	89%	86%
Black	6%	8%
Hispanic	2%	2%
Asian	2%	2%
Native American	0.1%	0.2%
Pacific Islander/other/unknown	1%	2%
Age, mean (standard deviation)	79 (6.1)	76 (6.2)

Abbreviations: PD = Parkinson disease; PM_2.5_ = particulate matter <2.5 micrometers in diameter.

a All Medicare beneficiaries with incident PD or without PD in 2009 who met basic criteria; methods and sample are described in Silver et al. 2020.^43^ Excludes 19,144 cases and 4,693,525 controls without geocodable zip+4 data for residence 2 years before PD diagnosis or the control reference date and 3,140 cases and 740,179 controls without PM_2.5_ data at the geocoded location in the United States.

b Percentage excludes 47 controls with unknown sex.

**Figure 1:**
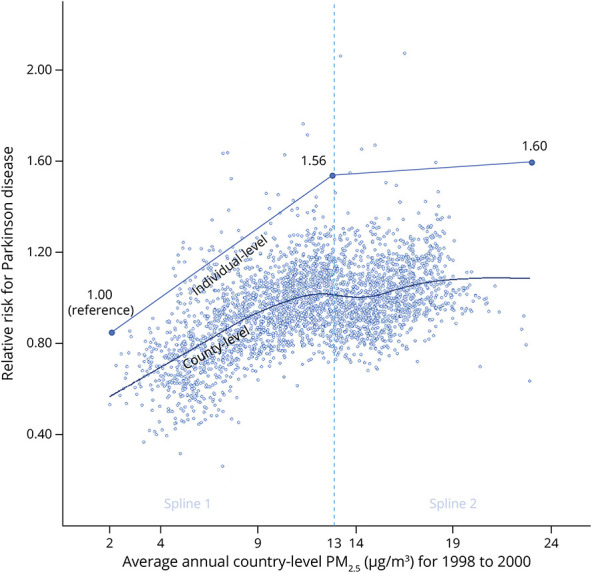
PM2.5 Exposure and Risk of PD Among US Medicare Beneficiaries in 2009 The relationship between individual-level (zip+4) PM_2.5_ exposure and risk of PD among US Medicare beneficiaries in 2009 was well-described by a logistic regression model with 2 linear splines with a knot at 13 μg/m^3^ PM_2.5_. The adjusted relative risk of PD was 1.56 (95% CI 1.47–1.66) when comparing beneficiaries exposed to 12.93 μg/m^3^ of PM_2.5_ (the median of PM_2.5_) with beneficiaries exposed to 2.16 μg/m^3^ of PM_2.5_ (the lowest level of PM_2.5_ and the reference group for the individual-level line in the plot). The adjusted relative risk of PD was 1.60 (95% CI 1.51–1.70) when comparing beneficiaries exposed to the highest PM_2.5_ with this same reference group. A locally weighted scatterplot smoothing curve between county-level mean PM_2.5_ for the smoothed, standardized county-PD relative risk (with all other counties in the contiguous United States as the reference group). Each dot represents a county.

We found a positive association between PM_2.5_ and PD where the RR for PD was 11%–28% higher in each of the 9 upper deciles of PM_2.5_ relative to the lowest decile (Table 2). The association was strictly linear in the lower 5 deciles up to at least 13 μg/m^3^ and then began to weaken as differences in PM_2.5_ levels became more similar across deciles. Nonetheless, risk continued to increase generally, with exception of the highest 2 deciles, resulting in a significant trend overall (*p*_trend_ < 0.0001) (Table 2). When fitting a model with 2 linear splines, the model with the lowest (best) Akaike information criterion (814702.9) incorporated a knot at 13 μg/m^3^ with 4.2% greater risk of PD for each additional μg/m^3^ of PM_2.5_ up to this level and then a weak, nonsignificant increase with increasing PM_2.5_ levels thereafter resulting in a plateau (Table 2, overall PM_2.5_-PD association *p* < 0.0001). The spline model was sensitive to knot placement, with 2 significant positive splines observed when the knot was at 12 μg/m^3^ (RR = 1.048, 95% CI 1.041–1.055 per μg/m^3^ of PM_2.5_ up to this level and 1.007, 95% CI 1.003–1.012 per μg/m^3^ of PM_2.5_ thereafter, Akaike information criterion 814710.6), for example, but equally good yet contrasting models with a knot at either 14 or 15 μg/m^3^. A simple linear model yielded a higher (poorer) Akaike information criterion (814770.9), and a likelihood ratio test confirmed a poorer fit relative to our best spline model (*p* < 0.0001). Based on the best spline model, the PD RR was 1.56 (95% CI 1.47–1.66) when comparing beneficiaries exposed to 12.93 μg/m^3^ of PM_2.5_ (median PM_2.5_) with beneficiaries exposed to 2.16 μg/m^3^ of PM_2.5_ (minimum PM_2.5_). With adjustment for 6 medical conditions associated with PM_2.5_ that might facilitate PD diagnosis, the RR was 1.43 (95% CI 1.35–1.51). Adjustment for socioeconomic variables, access to neurologists, and pesticide use did not affect the RR for PM_2.5_ and PD (eTable 1, links.lww.com/WNL/D163). Airborne trichloroethylene also did not confound the PM_2.5_-PD association (Table 2). In addition, the results were unchanged when Lewy body dementia was included in the case definition. We found no evidence that the PM_2.5_-PD association differed by age (***p***_***interaction***_ = 0.77). The pattern of association was the same in men and women, but with slightly stronger associations in women for both splines (***p***_***interaction***_ = 0.001). The counties with average annual PM_2.5_ levels that fell within Spline 1 were largely in the Western half of the United States (Figure 1). However, the results were fairly similar in urban areas, suburban areas, small towns, and rural areas, with significant RRs ranging from 1.040 to 1.047 per μg/m^3^ of PM_2.5_ up to 13 μg/m^3^ and then relatively flat thereafter for each of land use type (likelihood ratio ***p***_***interaction***_ = 0.69).

**Table 2:**
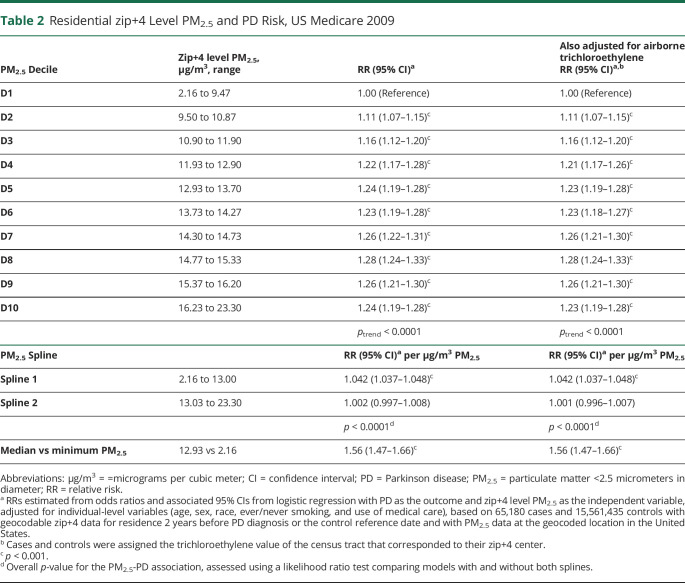
Residential zip+4 Level PM_2.5_ and PD Risk, US Medicare 2009

PM_2.5_ Decile	Zip+4 level PM_2.5_, μg/m^3^, range	RR (95% CI)^a^	Also adjusted for airborne trichloroethylene RR (95% CI)^a,b^
D1	2.16 to 9.47	1.00 (Reference)	1.00 (Reference)
D2	9.50 to 10.87	1.11 (1.07–1.15)^c^	1.11 (1.07–1.15)^c^
D3	10.90 to 11.90	1.16 (1.12–1.20)^c^	1.16 (1.12–1.20)^c^
D4	11.93 to 12.90	1.22 (1.17–1.28)^c^	1.21 (1.17–1.26)^c^
D5	12.93 to 13.70	1.24 (1.19–1.28)^c^	1.23 (1.19–1.28)^c^
D6	13.73 to 14.27	1.23 (1.19–1.28)^c^	1.23 (1.18–1.27)^c^
D7	14.30 to 14.73	1.26 (1.22–1.31)^c^	1.26 (1.21–1.30)^c^
D8	14.77 to 15.33	1.28 (1.24–1.33)^c^	1.28 (1.24–1.33)^c^
D9	15.37 to 16.20	1.26 (1.21–1.30)^c^	1.26 (1.21–1.30)^c^
D10	16.23 to 23.30	1.24 (1.19–1.28)^c^	1.23 (1.19–1.28)^c^
		*p*_trend_ < 0.0001	*p*_trend_ < 0.0001

PM_2.5_ Spline		RR (95% CI)^a^ per μg/m^3^ PM_2.5_	RR (95% CI)^a^ per μg/m^3^ PM_2.5_
Spline 1	2.16 to 13.00	1.042 (1.037–1.048)^c^	1.042 (1.037–1.048)^c^
Spline 2	13.03 to 23.30	1.002 (0.997–1.008)	1.001 (0.996–1.007)
		*p* < 0.0001^d^	*p* < 0.0001^d^
Median vs minimum PM_2.5_	12.93 vs 2.16	1.56 (1.47–1.66)^c^	1.56 (1.47–1.66)^c^

Abbreviations: μg/m^3^ = =micrograms per cubic meter; CI = confidence interval; PD = Parkinson disease; PM_2.5_ = particulate matter <2.5 micrometers in diameter; RR = relative risk.

a RRs estimated from odds ratios and associated 95% CIs from logistic regression with PD as the outcome and zip+4 level PM_2.5_ as the independent variable, adjusted for individual-level variables (age, sex, race, ever/never smoking, and use of medical care), based on 65,180 cases and 15,561,435 controls with geocodable zip+4 data for residence 2 years before PD diagnosis or the control reference date and with PM_2.5_ data at the geocoded location in the United States.

b Cases and controls were assigned the trichloroethylene value of the census tract that corresponded to their zip+4 center.

c *p* < 0.001.

d Overall *p*-value for the PM_2.5_-PD association, assessed using a likelihood ratio test comparing models with and without both splines.

**Figure 2:**
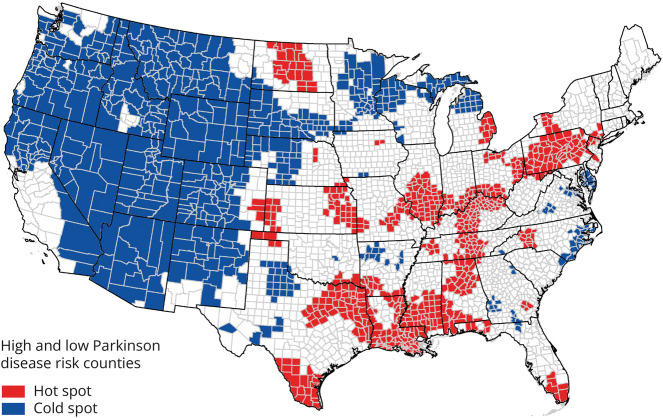
Parkinson Disease Risk Hot and Cold Spots Areas of high (hot spots) and low (cold spots) risk of PD among Medicare beneficiaries in the contiguous United States in 2009. Hot and cold spots were identified using univariate LISA analyses and county-PD relative risks that account for age, sex, race, smoking, health care utilization, and spatial dependency. LISA = local indicator of spatial association; PD = Parkinson disease.

**Figure 3:**
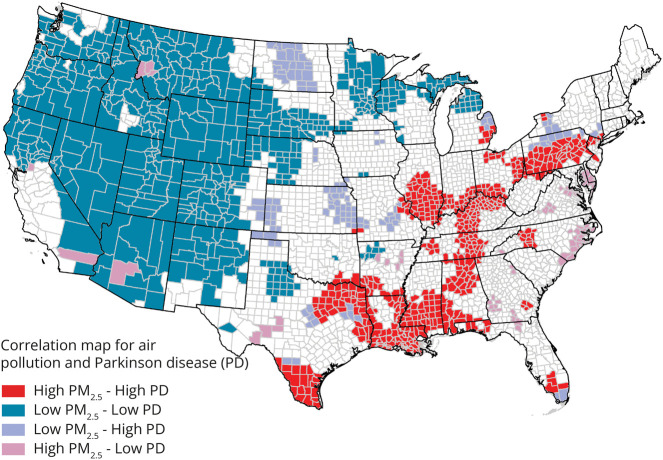
Spatial Correlation Between PM_2.5_ and Parkinson Disease Risk Cluster map showing how PD hot and cold spots among US Medicare beneficiaries in 2009 overlay with PM_2.5_ hot and cold spots in 1998–2000. Clusters were identified with bivariate LISA using (1) relative risks that account for age, sex, race, smoking, health care utilization, and spatial dependency and (2) average annual PM_2.5_. The high-high category represents counties where risk of PD and PM_2.5_ exposure are both high relative to means. The low-high and high-low clustering categories describe spatial outliers where a low-high county is one with below-average PD risk and above-average PM_2.5_. The low-low category represents counties where PD risk and exposure are both less relative to their means. LISA = local indicator of spatial association; PD = Parkinson disease; PM_2.5_ = particulate matter <2.5 micrometers in diameter.

**Figure 4:**
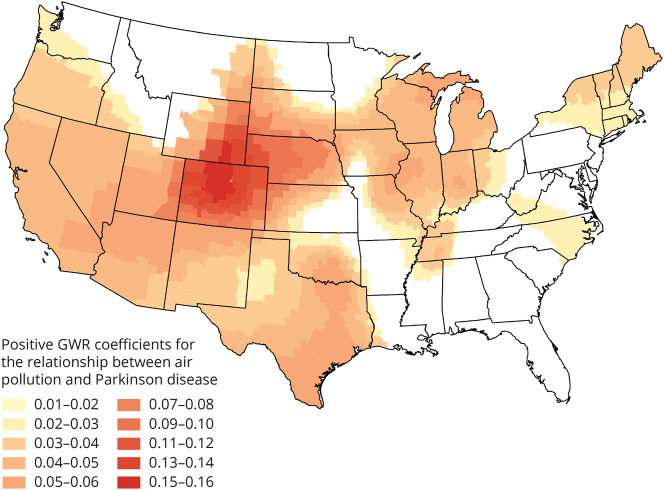
Strength of Positive Associations Between PM_2.5_ and Parkinson Disease Risk GWR coefficient map showing location and strength of significant positive coefficients for the association between county-level average annual PM_2.5_ and Parkinson disease relative risk in the United States that account for age, sex, race, smoking, health care utilization, and spatial dependency. Each coefficient represents the absolute difference in the relative risk with each additional decile of PM_2.5_ exposure. The strongest associations appear in Colorado and Wyoming, which have relatively low risk of Parkinson disease and relatively low PM_2.5_ levels compared with the rest of the nation. This pattern of coefficients is an artifact of a possible ceiling effect in the Mississippi-Ohio River Valley, which has above-average Parkinson disease risk and some of the highest PM_2.5_ levels in the nation. These patterns of coefficients are consistent with the observed positive association between PM_2.5_ and PD nationwide overall that is stronger at lower PM_2.5_ levels than higher PM_2.5_ levels. GWR = geographically weighted regression; PM_2.5_ = particulate matter <2.5 micrometers in diameter.

In univariate LISA (hot and cold spot) analysis, we found moderate clustering of similar values for PD risk across the contiguous United States (*Global Moran's I* value of 0.500, *p < 0.*05). The associated LISA map revealed an S-shaped pattern of high PD risk in the Mississippi-Ohio River Valley and a PD cold spot in a large portion of the Western part of the nation (Figure 2). PD risk was 19% greater in the Mississippi-Ohio River Valley hot spot compared with the rest of the nation. Bivariate LISAs revealed local spatial correlation characterized by high-high clusters (counties with above average PD risk and above average PM_2.5_) and low-low clusters (counties with below average PD risk and below average PM_2.5_) in the above respective areas (Figure 3). Several other smaller high-high or low-low areas and only a small number of discordant areas were observed. The Monte Carlo test of spatial variability further revealed spatial variation in the local parameter estimates for PM_2.5_ as a PD risk factor (*p* = 0.0001). GWR identified specific clusters of counties where the positive association between PM_2.5_ and PD risk was the strongest (Figure 4). The strongest positive coefficients formed a cluster of 51 counties within our PD cold spot in the mountainous regions of Colorado and Wyoming, where the PM_2.5_-PD RR increased, in absolute terms, by approximately 0.15–0.16 per decile of PM_2.5_ exposure. Although much weaker, we also identified a positive association between PD and PM_2.5_ in our PD hot spot: For 118 counties North of the Mississippi-Ohio river confluence (in Indiana, Ohio, Illinois, and Missouri), the RR of PD increased by approximately 0.03–0.04 with each additional decile of PM_2.5_ exposure. Four regions of the country demonstrated null or inverse associations between PM_2.5_ and PD. These included the North Dakota-Minnesota border; parts of the Mid-Atlantic; South Atlantic; and a region that spans part of Washington State, Idaho, and Montana (eFigure 1, links.lww.com/WNL/D162).

## Discussion

In this multimethod study investigating the nationwide patterns of PD risk and its relation to PM_2.5_, we found an S-shaped pattern of high PD risk (hot spot) in the Mississippi-Ohio River Valley and low PD risk (cold spot) in the Western United States. This geographic pattern aligns broadly with our prior study of incident PD^21^ while providing a more refined pattern. Our bivariate LISA correlation map for PD and PM_2.5_ closely overlapped with our PD risk map, in that PD hot spots generally aligned with PM_2.5_ hot spots and PD cold spots generally aligned with PM_2.5_ cold spots. Although bivariate LISA maps are exploratory and cannot alone confirm relationships at the nation level, we confirmed a PM_2.5_-PD association nationally. In our regression analysis using individual-level data, the association between PD and PM_2.5_ was linear up to at least 13 μg/m^3^ PM_2.5_. At the highest levels of PM_2.5_, the relationship between PM_2.5_ and PD appeared to plateau, but the overall association remained positive. Our GWR results also suggested a possible ceiling effect as the association weakened in the Mississippi-Ohio River Valley where some of the highest levels of PM_2.5_ in the nation overlay regions with some of the highest PD risk in the nation. Although the reason for the plateau is unclear, several studies report effect estimates consistent with a similar plateau.^12-14,16^ It is more important that the robust dose-response association at the lower levels of PM_2.5_ potentially has substantial public health relevance. Recently, the US Environmental Protection Agency proposed to revise the primary (health-based) annual PM_2.5_ standard level from 12 μg/m^3^ to 9–10 μg/m^3^ because of growing evidence of health effects at levels lower than the previous regulatory standard.^44^ Our study provides important additional evidence supporting this proposal. Previous studies,^8,16,20^ but not all,^15^ demonstrated clear linear associations at this lower range of PM_2.5_ levels while the observed range of PM_2.5_ levels or analytic approaches in all or most of the remainder could have obscured these associations. Therefore, our study provides insights that strengthen the interpretation of the broader literature as generally consistent with PM_2.5_ as an exposure that increases risk of PD.

Key strengths of our study are that we used large, population-based data and were restricted to incident disease, aspects particularly important for geographic studies designed to inform disease etiology. Many prior studies used other PD-related outcomes that might be affected by PD progression or survival, not just risk. We chose to maximize sensitivity of identification of incident PD to ensure that our work was particularly resilient to selection bias.^26^ Selection bias might occur in other studies with more restrictive definitions of incident PD if, for example, PD cases from higher vs lower PM_2.5_ areas are less able to access neurologist care or survive long enough to begin anti-parkinsonian medications, that is, be ascertained as a case. In addition, our results are less vulnerable to biases from exposure measurement error because our zip+4 PM_2.5_ data serves as a stronger proxy for individual-level exposure compared with environmental studies that use broader units of geography-based exposure assignment. Furthermore, our study leveraged new and innovative geographic information system methods. Geographic approaches offer insight beyond multilevel (cluster) regression modeling by enabling investigators to adjust for confounders within their maps and smooth extreme values by taking into account neighboring values, thus refining local patterns of disease. Another important strength is that, within the context of our population-based sample, we also aimed to be as inclusive as possible with not only case ascertainment but also study eligibility, applying only scientifically essential criteria. This allowed us to include minority, poor, and rural populations—and the PD cases that arise within these populations—that are often missed in epidemiologic studies of PD.^45^

We found a clear linear dose-response relation between PM_2.5_ and PD up to at least 13 μg/m^3^, and others have also observed a linear association across a similar range of PM_2.5_ levels as in our study.^8,16,20^ Two of these studies found a similar magnitude of effect, estimating ∼50% greater risk of PD when comparing ∼13 μg/m^3^ of PM_2.5_ with the lowest levels of exposure of ∼2 μg/m^3^.^8,16^ Within this approximate PM_2.5_ range, the third, smaller study with local air monitoring data reported a linear association of ∼50% greater magnitude.^20^ If the PM_2.5_-PD association is causal, future studies investigating the association between PD and PM_2.5_ subfractions might demonstrate an even greater effect size and might provide additional insights to PD etiology. A multicountry study in Europe confirms the importance of considering the subfractions of PM_2.5_.^8^

Accordingly, one potential explanation for the heterogeneity in PM_2.5_-PD associations across the many studies conducted to date^6-20^ is that PM_2.5_ is not a homogenous exposure, but rather a mixture of chemical components that varies by source. PM_2.5_ can be produced from a variety of sources, including industrial emissions,^8^ motor vehicle traffic,^19^ and farming practices.^10^ PM_2.5_ may, therefore, have varied effects on the development of neurodegenerative disease depending on the chemical composition of PM_2.5_ in different regions. Of particular relevance, studies have identified heavy metals within PM_2.5_ in the Ohio River Valley, including manganese and zinc linked to iron and steel manufacturing.^46,47^ In addition to the type of, and proximity to, emission sources, the physical environment may also influence levels of relevant PM_2.5_ components and, hence, health effects. For example, the S-shaped pattern of high risk in the Mississippi-Ohio River Valley follows the low-elevation valleys along the Appalachian Mountains, suggesting a potential role of regional wind patterns and topography in the relationship between geographic exposures such as PM_2.5_ and PD. Our methods allowed for the detection of differential effects across the subregions of the United States. In most regions, we detected a strong positive relationship between PM_2.5_ and PD, whereas in a few regions, there was no detectable effect or there was evidence of inverse associations. These results might inform efforts to identify natural and built environment conditions that could provide some protections against air pollution, including specific climates and urban layouts. However, we cannot rule out the possibility that regional differences could be partly due to gene-environment interaction^48^ and/or interactions with various nongenetic factors including diet.^49^ The differential effects across regions emphasize the value of taking a geographic approach in large studies. Thus, we encourage the application of this approach in other parts of the world, including densely populated regions in South Asia where PM_2.5_ levels can reach ∼60 μg/m^3^ on average^50^ (compared with 14 μg/m^3^ for the contiguous United States at the time of our study).

There are several limitations of this work. Our geographic analyses relied on aggregate data subject to the ecological fallacy. Nevertheless, regression based on individual-level outcome data corroborated our county-level geographic findings. At the same time, we acknowledge that the separate issue of exposure measurement error (likely nondifferential) remains to some extent. Any PM_2.5_ exposure model is imperfect, even the one we used, which achieved a cross-validated R^2^ of 0.89.^28^ In addition, we linked the objective PM_2.5_ estimates from this model using zip+4 rather than exact addresses. Another limitation of Medicare data is the restriction on coverage to patients 65 years and older, although the PM_2.5_-PD association did not differ by age. We excluded patients with Medicare Advantage plans from analyses because these plans do not report claims to Medicare, but during the study years, they only represented a quarter of beneficiaries,^25^ so we anticipate that our results remain generalizable. We also excluded a small percentage of potential cases whose PD incident status was uncertain because of the diagnosis of Lewy body dementia and/or atypical parkinsonism. However, the results of our sensitivity analysis found no difference in the results when Lewy body dementia was included in our case definition. Our results also assume that beneficiaries were nonmobile during the 10 years before diagnosis; we acknowledge that our methods were unable to capture early-life exposures that might be relevant. That said, the overwhelming majority of beneficiaries in our study were nonmobile for at least the 5 years of data which were available to us. Owing to the long PD prodromal period,^25^ we applied as much exposure lagging as possible and used PM_2.5_ estimates from up to 10 years before diagnosis. The true period of relevant exposure may be longer, but we were unable to extend our exposure period or explore time windows of exposure given that earlier years of residence data were not available. We also acknowledge that the association between PM_2.5_ and PD could result, at least in part, from a correlated, environmental exposure, but we ruled out confounding by both pesticides and trichloroethylene. In addition, while we found no evidence that the PM_2.5_-PD association was stronger in men than women, we cannot rule out confounding by occupational exposures. Finally, we also note that the validity of our data on PD diagnosis requires individuals to obtain medical care and relies on competent diagnoses and data entry by health care providers and staff. A prior study suggested that the case identification method we used in our study had 82.7% sensitivity and 99.7% specificity among primary care patients,^26^ and through our design and analysis choices, we sought to minimize the potential for selection bias and confounding by use of medical care. Despite limitations, our study advances current knowledge of PM_2.5_ in relation to PD and demonstrates the utility of using a multimethod geographic approach for investigating environmental risk factors.

Using state-of-the-art geographic analytic techniques, we identified strong regional associations between PM_2.5_ and PD in the United States. A deeper investigation into the subfractions of PM_2.5_ in those regions may provide insight into PD risk factors.

## Appendix Authors

**Table:**

Name	Location	Contribution
Brittany Krzyzanowski, PhD	Barrow Neurological Institute, Phoenix, AZ	Drafting/revision of the manuscript for content, including medical writing for content; major role in the acquisition of data; study concept or design; analysis or interpretation of data
Susan Searles Nielsen, PhD	Washington University in St. Louis, MO	Drafting/revision of the manuscript for content, including medical writing for content; major role in the acquisition of data
Jay R. Turner, DSc	Washington University in St. Louis, MO	Drafting/revision of the manuscript for content, including medical writing for content
Brad A. Racette, MD	Barrow Neurological Institute, Phoenix, AZ	Drafting/revision of the manuscript for content, including medical writing for content; major role in the acquisition of data

## Glossary

μg/m^3^: micrograms per cubic meter
CI: confidence interval
GWR: geographically weighted regression
LISA: local indicator of spatial association
PD: Parkinson disease
PM_2.5_: particulate matter <2.5 micrometers in diameter
R-INLA: Laplace approximation method for Bayesian inference in R-project
RR: relative risk
U.S.: United States
